# Effects of Betaine Aldehyde Dehydrogenase-Transgenic Soybean on Phosphatase Activities and Rhizospheric Bacterial Community of the Saline-Alkali Soil

**DOI:** 10.1155/2016/4904087

**Published:** 2016-09-04

**Authors:** Ying Nie, Da-qing Wang, Guang Zhao, Song Yu, Hong-yan Wang

**Affiliations:** ^1^College of Resources and Environment, Northeast Agricultural University, Harbin 150030, China; ^2^Heilongjiang State Farms Institute of Economy, Harbin 150090, China; ^3^School of Chemical & Environmental Engineering, Liaoning University of Technology, Jinzhou, Liaoning 121001, China; ^4^College of Agriculture, Heilongjiang Bayi Agricultural University, Daqing, Heilongjiang 163319, China

## Abstract

The development of transgenic soybean has produced numerous economic benefits; however the potential impact of root exudates upon soil ecological systems and rhizospheric soil microbial diversity has also received intensive attention. In the present study, the influence of saline-alkali tolerant transgenic soybean of betaine aldehyde dehydrogenase on bacterial community structure and soil phosphatase during growth stages was investigated. The results showed that, compared with nontransgenic soybean as a control, the rhizospheric soil pH of transgenic soybean significantly decreased at the seedling stage. Compared to HN35, organic P content was 13.5% and 25.4% greater at the pod-filling stage and maturity, respectively. The acid phosphatase activity of SRTS was significantly better than HN35 by 12.74% at seedling, 14.03% at flowering, and 59.29% at podding, while alkaline phosphatase achieved maximum activity in the flowering stage and was markedly lower than HN35 by 13.25% at pod-filling. The 454 pyrosequencing technique was employed to investigate bacterial diversity, with a total of 25,499 operational taxonomic units (OTUs) obtained from the 10 samples. Notably, the effect of SRTS on microbial richness and diversity of rhizospheric soil was marked at the stage of podding and pod-filling. Proteobacteria, Acidobacteria, and Actinobacteria were the dominant phyla among all samples. Compared with HN35, the relative abundance of Proteobacteria was lower by 2.01%, 2.06%, and 5.28% at the stage of seedling, at pod-bearing, and at maturity. In genus level, the relative abundance of Gp6,* Sphingomonas* sp., and GP4 was significantly inhibited by SRTS at the stage of pod-bearing and pod-filling.

## 1. Introduction

Genetically modified (GM) plants have been widely commercialized throughout the world, and more than 160 million hectares have been released for cultivation of GM plants [1]. In the meantime, the potential risk of horizontal gene transfer to the environment has received intensive attention from many scientists, who have studied, for example, the impact of GM on soil processes and microbial community diversity of rhizospheric soil [2]. Transgenic soybean is one of the most important GM plants, and its planting area reached about 70 million hectares in 2009 [3]. The betaine aldehyde dehydrogenase- (BADH-) transgenic soybean is genetically modified by betaine aldehyde dehydrogenase, which possesses a tolerance to saline-alkali soils. The BADH gene has a resistance to saline-alkali stress and drought stress and has transformed many crops including rice, maize, and potato, among others [4].

According to FAO (Food and Agriculture Organization) statistics, there are more than 900 million hectares of saline-alkaline soils, mainly in arid and semiarid regions of the world [5, 6]. Songnen Plain is one of the three primary contiguous saline-alkaline soil regions of the world, with an area of 3.73 million hectares in northeastern China. Due to a high pH, high sodium absorption ratio, poor soil structure, and the low nutrient level of saline-alkaline soils, crop productivity and therefore economic development are intensively restricted. Therefore, soybean which is moderately tolerant of salinity is a feasible crop for Songnen Plain, as it is rich both in nutritional value and in great potential economic benefits. Although BADH-transgenic soybean has a tolerance to saline-alkaline stress, there is potential for root exudates to alter the soil chemistry process and soil microbial functions. In many countries, the pressure to improve crop production to satisfy human demands leads to approval of policies of planting saline-alkali tolerant plants, without then addressing the influence of these crops on the soil ecological process [7, 8].

Most studies concerning soil biological safety of transgenic plants are focused on the diversity of soil microbial community, such as soil enzyme activities, root exudates, and chemical processes of rhizosphere soil [9–11]. The soil ecosystem is important for nutrition cycle, surrounding minerals, and energy conversion and exchange. However, the potential risks of GM plants to ecological, environmental, and human health have become a public concern in recent years [12]. The rhizosphere is considered the most influential element of soil formation, which is intensively influenced by root secretions and associated bacterial diversity and functions [13]. Soil microbial enzyme activities, especially for functional groups of bacteria involved in C, N, and P cycling, are stimulated in the rhizosphere [14]. Biological and biochemical properties of soil have often been proposed as early sensitive indicators of soil ecological stress and other environmental changes. Generally, dehydrogenase and phosphatase enzyme activities in soil are closely related to the build-up of organic matter and provide sensitive information on the microbial activity of soil, which is thought to reflect the total range of oxidative activity of soil microflora, and consequently may be a good indicator of microbiological activity [15]. The types of root exudates and residual plant materials have an important impact on microbials residing in the rhizosphere. Any change in the quality and quantity of root exudates could potentially modify the composition and activity of soil microbials and may cause changes in both deleterious and beneficial microorganisms. This supports the idea that genetic modification of plants or microorganisms may cause shifts in microbial communities [16]. The BADH-transgenic soybean will not only greatly increase soybean production, but also promote the biological improvement for saline-alkaline soil in Songnen Plain. Nevertheless, few environment assessments have been reported for transgenic soybean expressing the BADH gene under field conditions.

Next-generation sequencing of 454 pyrosequencing technology is a powerful approach for studying bacterial diversity in various environment samples, such as soil and wastewater [17, 18]. In this study, the growth stage-related dynamics of rhizospheric soil pH and phosphatase activities were evaluated. Moreover, 454 pyrosequencing technology was employed to investigate potential effects on the bacterial composition and diversity in the rhizosphere of transgenic soybean lines expressing the spinach betaine aldehyde dehydrogenase gene, compared to the nontransgenic parental cultivar at different plant development stages under field conditions of saline-alkaline soil in Songnen Plain. The results from this study will contribute to the assessment of environmental risks of transgenic crops to the soil ecosystem.

## 2. Materials and Methods

### 2.1. Study Site and Plants

The transgenic plants and control plants were grown on Anda farm at 46°01′ north and 124°55′ longitude located in Songnen Plain, Northeast China. The experimental soil is operated at a long-term (25 years) unfertilized and uncropped treatment of saline-alkali plot, and the plot was subdivided into 12 subplots (10 m × 6.5 m each). The trial soil belongs to the soda-saline soil, and initial characteristics of the surface were as follows: subclass: 38.9% sand, 39.7% silt, and 21.3% clay; pH: 8.34 ± 0.06; total organic carbon: 3.92 ± 0.04 g kg^−1^; total nitrogen (N): 1.16 ± 0.03 g kg^−1^ and 45.35 ± 0.53 mg/kg of alkali-hydrolysable N; total phosphorus (P): 0.62 ± 0.01 g kg^−1^ and 25.79 ± 0.33 mg/kg of available P; 148.28 ± 2.06 mg/kg of available potassium.

The transgenic soybeans used in the experiments were* BADH*-transgenic soybean (SRTS) and non-*BADH*-transgenic soybean (HN35), which were provided by Heilongjiang Academy of Agricultural Sciences, Harbin, China.

### 2.2. Experimental Design and Soil Sampling

The rhizosphere soil samples were collected at five different stages of plant growth: 25, 50, 75, 100, and 125 days after sowing (DAS). At every sampling stage, five randomly chosen plants per subplot were removed and the adhering soil was placed in sterile plastic bags (≈2.5 kg of moist soil) from six replicate plots of soybean in the same day; after removal of roots the soil samples were homogenized and were pooled together in order to obtain a representative sample for library construction. The samples were transported to the laboratory in dry ice chests within 2 hours. Then, one portion of the composite soil was used for the enzyme activity measurements, while other smaller amounts of the soil (10 g) were separated, stored in centrifuge tubes, and kept at −20°C in the dark until DNA extraction and further processing.

### 2.3. Determination of Soil pH and Phosphatase Enzyme Activity

The rhizospheric soil pH was determined using pH meter (320-S, Mettler-Toledo Instruments, Co., Ltd., Shanghai, China) with 1 : 1 ratio water/soil at room temperature (25°C). Acid phosphatase activity and alkaline phosphatases activity derived from rhizospheric soil were measured according to the method by Tabatabai [19]. All of the values were calculated in triplicate.

### 2.4. Microbial Community Analysis

#### 2.4.1. DNA Extraction from Rhizosphere Soil Samples

Soil genomic DNA was extracted directly from the rhizosphere soil samples using the soil DNA kit (Omega Biotek, Inc., Norcross, GA, USA) according to the manufacturer's instructions. The amount, quality, and purity of the extracted soil DNA were checked by electrophoresis in 1% agarose gels in 1x TBE buffer, run for 1 h at 90 V, and stained with ethidium bromide, upon exposure to UV.

#### 2.4.2. PCR Amplification and Pyrosequencing

PCR was performed using the primers 341f(5′-GCCTCCCTCGCGCCAT-CAGNNNNNNNCCTACGGGAGGCAGCAG-3′) and 534r(5′-*GCCTTGCCAGCCCGCTCAG* NNNNNNNATTACCGCGGCTGCTGG-3′), in which the italicized sequence is that of a 454 Life Sciences® primer and the underlined sequence is a barcode sequence tag. Amplification was performed in a 50 *μ*L reaction volume, which contained 100 ng genomic DNA, 1x reaction buffer, 3 mM MgCl_2_, 20 pmol each primer, 0.4 mM (each) dNTP, and 1.25 U of Taq DNA polymerase. The amplification conditions consisted of an initial denaturation step of 94°C for 3 min, followed by 30 cycles of 94°C for 30 s, 56°C for 30 s, and 72°C for 45 s, and a final elongation step at 72°C for 3 min. All of the PCR reactions were conducted using a GeneAmp PCR System 9700 (Applied Biosystems, Foster City, CA, USA).

#### 2.4.3. Statistical and Bioinformatics Analysis

The microbial sequences were sorted into each sample batch using the barcode tag in the Pipeline Initial Process at the RDP, and the RDP classifier (http://rdp.cme.msu.edu/classifier/classifier.jsp) was applied to classify the sequence data from genus to phylum at hierarchical levels [20]. Distance matrices and statistical parameters, including rarefaction curves, Shannon-Weaver indices, Simpson's diversity (1-D), coverage estimators (SACE), and Chao1 (SChao1), were calculated using Mothur software [21].

## 3. Results and Discussion

### 3.1. Dynamic Change of Rhizosphere Soil pH

**Figure 1:**
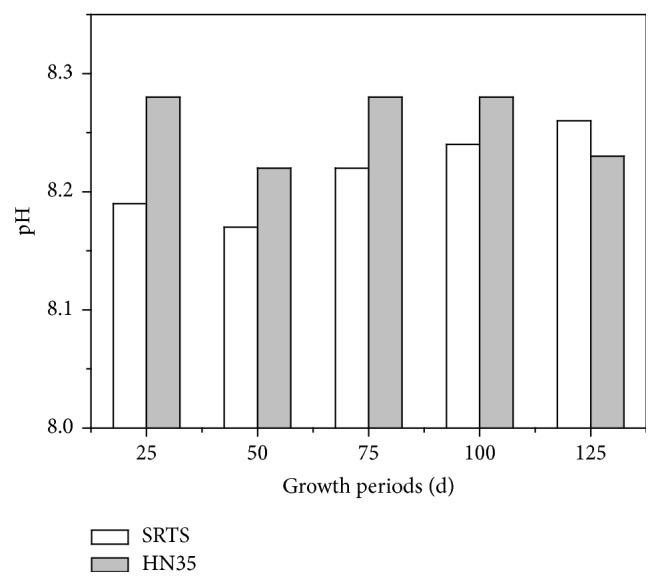
The pH of the rhizospheric soil of transgenic soybean and nontransgenic soybean at different growth stages.

The rhizospheric soil pH variation of transgenic soybean (SRTS) and nontransgenic soybean (HN35) exhibited similar trends during the whole growth period. Compared with HN35, the pH of SRTS was lower at 25, 50, 75, and 100 days after sowing time (Figure 1). The pH of SRTS (8.19) was significantly lower than for HN35 at seedling stage. However, with the growth of the soybean, the SRTS pH eventually exceeded that of HN35 at maturity stage, which might be due to the amount of root exudates produced. Therefore, more H^+^ secretion could be released by SRTS rhizosphere and cause pH to decrease under the stress of a saline-alkali and low phosphorus environment. The population of bacteria and the phosphorus availability of SRTS rhizospheric soil likely increased due to the decrease of pH at the seedling stage.

### 3.2. Effects of Transgenic Soybean on Organic P and Phosphatase Activities in Rhizospheric Soil

**Figure 2:**
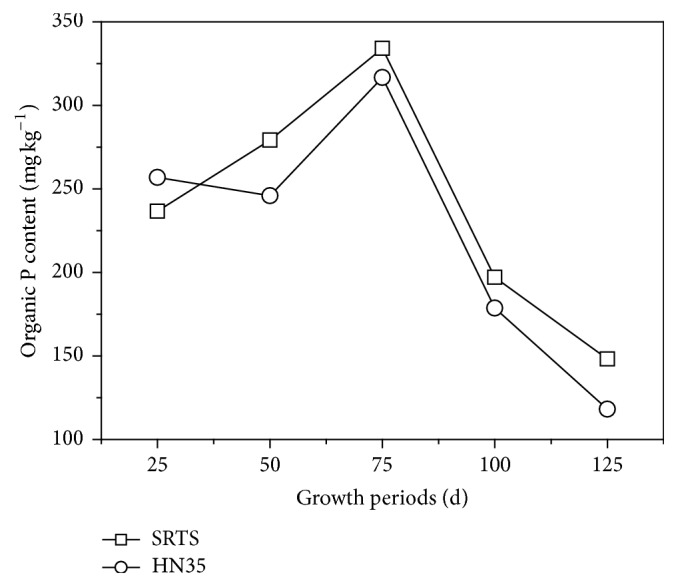
Organic P of the rhizospheric soil of transgenic soybean and nontransgenic soybean at different growth stages.

The organic P content in rhizospheric soil planted with SRTS and HN35 increased initially and then reached maximum content at pod-filling stage (Figure 2). The treatments of SRTS organic P were higher than for HN35 during growth periods but lower at the seeding stage. Compared to HN35, organic P was 13.5% and 25.4% greater at pod-filling stage and maturity stage, respectively. Organic P is significant for immobilization and mineralization of soil P. Due to the low inorganic P content of saline-alkali soil, mineralization of organic P was supplied for the plant. The results showed that mineralization of organic P was significantly influenced by the growth stages of transgenic soybean.

**Figure 3:**
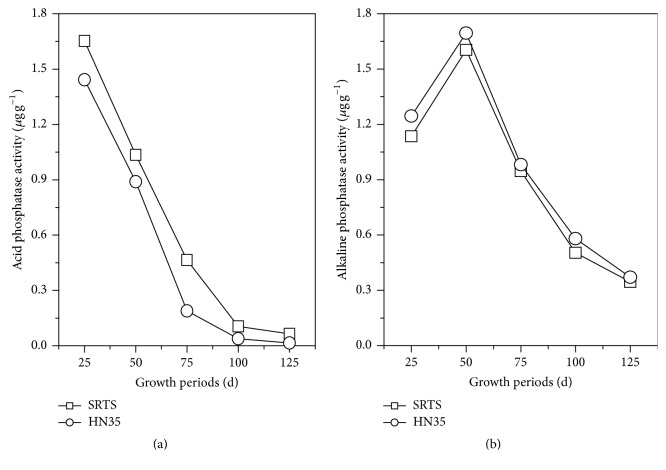
Activity of acid phosphatase (a) and alkaline phosphatase (b) in rhizospheric soil under transgenic soybeans and nontransgenic isolines during different growth stages.

The phosphatase activity controls the release of organic phosphorus and the biological validity of soil. Activity of acid phosphatase (a) and alkaline phosphatase (b) showed a similar trend in rhizospheric soil for SRTS and HN35 during different growth periods (Figure 3). The activity of acid phosphatases was highest at the seedling stage and gradually decreased with the growth of the soybean. The acid phosphatase activity of SRTS was significantly higher than HN35 at the seedling, flowering, and podding stages in rhizosphere soil, at 12.74%, 14.03%, and 59.29% higher, respectively. Therefore, the effect of SRTS on the activity of rhizosphere soil acid phosphatase was significantly influenced by changes in soybean growth stage. The activity of alkaline phosphatase achieved maximum effect in the flowering stage and decreased in the following growth stage. Compared with HN35, the effect of SRTS on rhizosphere soil alkaline phosphatase was markedly low and decreased by 13.25% in pod-filling stage.

### 3.3. Effects of BADH Soybean on Bacterial Communities Diversity in the Rhizospheric Soil

#### 3.3.1. Sequences Analysis of Pyrosequencing

**Figure 4:**
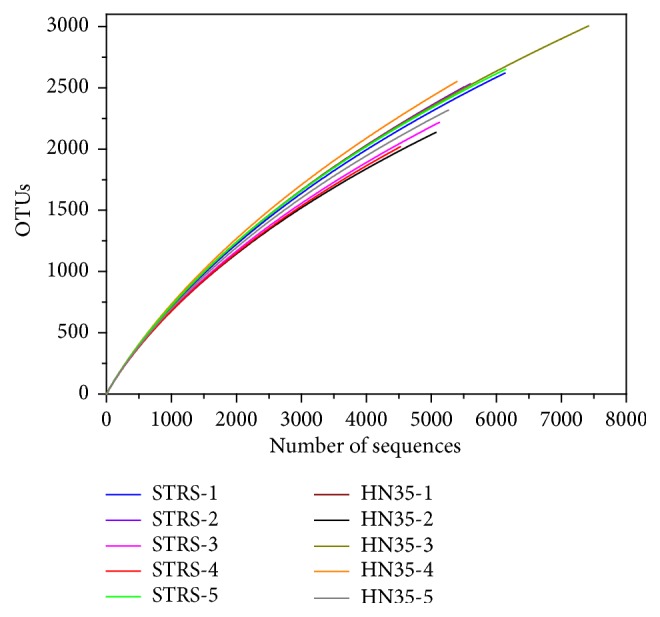
Rarefaction curves of sequences of 16S rRNA. The rarefaction curves from rhizospheric soil samples of transgenic soybeans and nontransgenic soybeans were calculated by 97% sequence similarity during different growth periods. Note: SRTS and BADH (1–5) mean 25 d, 50 d, 75 d, 100 d, and 125 d of different stages of soybean growth periods.

A total of 56,119 valid reads and 25,499 operational taxonomic units (OTUs) were obtained from the 10 samples of rhizospheric soil by 454 pyrosequencing analysis at 3% sequence similarity distance (Table 1). Rarefaction curves at 97% cut-off showed similar trends for all samples, yet none had reached saturation plateau (Figure 4). Despite examining nearly 5600 sequences of each sample, the ACE and Chao1 indicated high diversity in the bacterial community of soybean rhizospheric soil. Therefore, deeper sequencing may detect new bacterial phenotypes in soybean rhizospheric soil samples. The average sample achieved at 3% distance was estimated at 71.14% (Table 1). Notably, the effect of SRTS on richness and diversity of rhizospheric soil was marked in the podding and pod-filling stages. These changes in microbial diversity and enzyme activity between SRTS and HN35 during the growth stage could probably cause shifts in microbial structures and biological process.

#### 3.3.2. Overall Phylogenetic Analysis of Bacterial Communities

**Figure 5:**
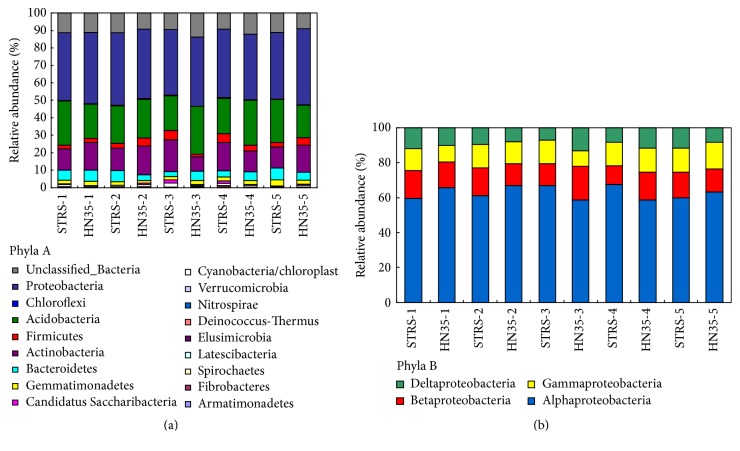
Taxonomic composition of different phyla for the sequences retrieved from rhizospheric soil samples of transgenic soybeans and nontransgenic soybeans during the whole growth stage based on the classification of partial 16S rRNA sequences of bacteria using RDP classifier. Phyla A: Bacteria phylum distribution; Phyla B: Proteobacteria classes distribution.

Many previous studies found that different varieties of plants could affect the microbial diversity of rhizospheric soil, yet little credible research has been conducted into whether transgenic plants can affect enzyme activities and the diversity of indigenous microbial populations [8, 22, 23]. In this study, the bacterial community analysis of rhizospheric soil for SRTS and HN35 at different growth stages was assessed using pyrosequencing technology, grouping the sequences at phylum. Of the classifiable sequences, 18 phyla were identified, and the dominant phyla were Proteobacteria, Acidobacteria, and Actinobacteria which were present in all samples (Figure 5(a)). Proteobacteria were the most dominant bacteria, which accounted for more than 38% of total sequences in all treatments. Compared with HN35, the relative abundance of Proteobacteria was 2.01%, 2.06%, and 5.28% lower in seedling, pod-bearing, and maturity stages, respectively. The abundance of phylum Acidobacteria was also clearly changed in transgenic soybeans of SRTS compared with nontransgenic soybeans of HN35, greater by 5.73% in seedling stage and 6.02% in the maturing period, yet the relative abundance of Acidobacteria was lower by 7.98% and 5.41% in pod-bearing and pod-filling stages, respectively. The obvious change of Actinobacteria occurred in the pod-bearing stage, where the relative abundance accounted for 18.14% in SRTS yet decreased to 8.36% in HN35. Furthermore, some less dominant bacteria were observed during different growth stages, such as Bacteroidetes, Firmicutes, Gemmatimonadetes, Cyanobacteria, Candidatus Saccharibacteria, and Nitrospirae.

**Figure 6:**
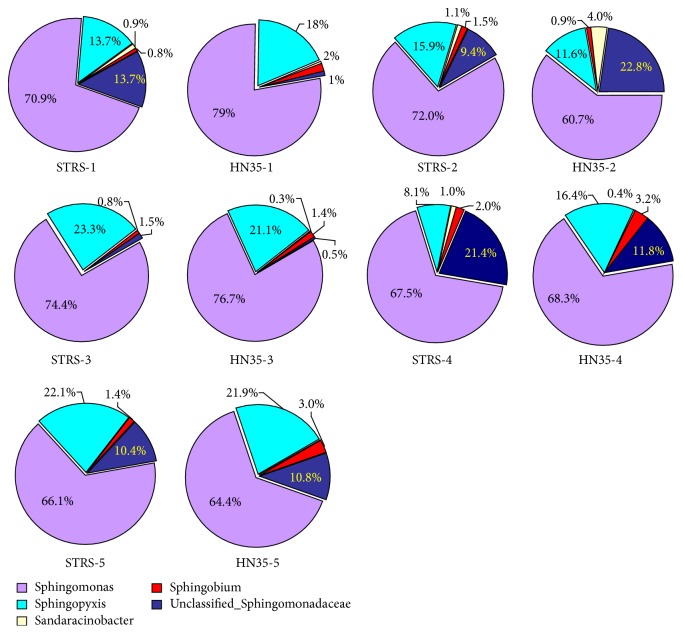
The relative abundance of Sphingomonadaceae family level distribution in each sample based on the classification of partial 16S rRNA sequences.

The most dominant class distribution of Proteobacteria observed was Alphaproteobacteria, which accounted for more than 58% of total sequences of all treatments (Figure 5(b)). The relative abundance of Alphaproteobacteria of SRTS was 6.15%, 6.04%, and 3.23% lower than in HN35 at seedling, flowering, and maturity stages, respectively; however, its abundance was 8.23% and 8.89% greater at pod-bearing and pod-filling stages. Clear changes in abundance of Betaproteobacteria were evident in pod-bearing and pod-filling stages compared with HN35, when relative abundance decreased by 6.75% and 5.28%, respectively. Notably, the class Deltaproteobacteria was 6.32% lower in SRTS than in HN35 at these growth stages and did not change significantly in other growth stages. In genus-level distribution of Sphingomonadaceae,* Sphingomonas *sp. dominated in all treatments, and* Sphingopyxis* sp. was the second predominant bacteria (Figure 6). It was clear that the relative abundance of STRS was 8.1% less than for HN35 at the seedling stage and 9.5% greater in the flowering stage.

In genus level, there were 32 genera found with a relative abundance exceeding 0.2% of total bacteria (Table 2). The dominant bacteria for STRS and HN35 were Gp6 (12.48% and 8.89%, resp.),* Sphingomonas *sp. (9.14%, 7.35%), Gp4 (5.37%, 2.93%),* Gemmatimonas *sp. (2.14%, 2.31%), Gp7 (1.68%, 1.25%),* Ohtaekwangia *sp. (1.52%, 1.80%), and Gp16 (1.24%, 1.04%) at seedling stage (relative abundance ≥ 1.0%). Notably, Gp6, Gp4, Gp7, and Gp16 belonged to Acidobacteria, dominating 20.77% and 14.11% of total sequences for STRS and HN35 at seeding stage. This was probably the reason that pH of SRTS rhizospheric soil was significantly lower than that for HN35 in seedling stage.

The relative abundance of genus Gp6,* Sphingomonas *sp., and GP4 was significantly inhibited by STRS compared with HN35. Gp6 was lower by 12.38% and 9.02% at pod-bearing stage and pod-filling stages; however, it was 6.03% greater in the maturing period. Genus* Sphingomonas *sp. accounted for 2.54% of bacteria in the pod-bearing stage, when minimum relative abundance was 5.75% lower than for HN35. The trend was opposite to that of the flowering stage, compared with HN35, when* Sphingomonas *sp. increased by 4.5%. The relative abundance of genus* Gemmatimonas *sp. was not significant between STRS and HN35 during the growth stage.

**Figure 7:**
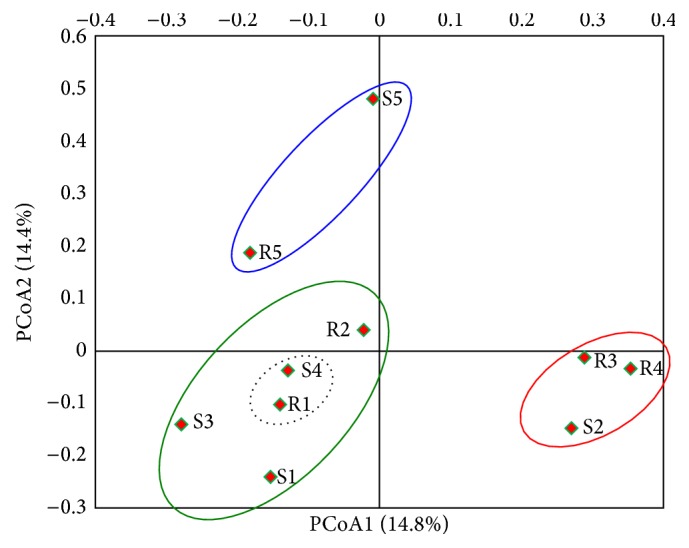
PCoA analysis of the bacteria of each sample at the 97% similarity of sequences based on UniFrac method. Principal component 1 and principal component 2 explained 14.8% and 14.4% of the total variations. Note: R1–R5 and S1–S5 mean 25 d, 50 d, 75 d, 100 d, and 125 d of STRS and NH35 of soybean at different growth stages.

The principal component analysis based on weighted UniFrac distance was used to reveal the relationships among the 10 samples at the 97% similarity of sequences (Figure 7). The principal component analysis showed that STRS-5 and NH35-5, STRS-1, NH35-4, STRS-2, NH35-1, and NH35-3 were grouped to the right of the principal component, which represented 14.8% of the total variation. STRS-3, STRS-4, and NH35-2 were grouped into a cluster near the principal component and accounted for 14.4%.

## 4. Conclusions

The potential risk of BADH-transgenic soybean to soil ecosystems has aroused great attention in recent years. In this study, the pH, organic P content, and phosphatase activity of rhizospheric soil were used to assess soil process changes between SRTS and HN35 over a growth period of time. Furthermore, the 454 pyrosequencing technique was employed to expose the soil microbial community. The results suggested that root exudates of BADH-transgenic soybean had a significant impact on phosphatase activity and bacterial diversity of rhizospheric soil at different growth stages. Compared to HN35, acid phosphatase activity of SRTS was significantly greater (59.29%) at the stage of podding, which accorded with organic P content variation, while alkaline phosphatase was markedly lower for SRTS by 13.25% at the stage of pod-filling. Bacterial richness and diversity were markedly influenced by SRTS at the stage of podding and pod-filling. Genus of Gp6,* Sphingomonas* sp., and GP4 were significantly inhibited by STRS at the stage of pod-bearing and pod-filling. Therefore, further research should focus on evaluating biological safety based on soil processes and change of soil microbial function.

## Figures and Tables

**Table 1:** Comparison of the estimated operational taxonomic unit (OTU) richness and diversity indices of the 16S rRNA gene for clustering at 97% identity as obtained from the pyrosequencing analysis.

Sample	^a^NS	^b^OTUs	Estimated OTU richness		Shannon	Coverage (%)
			ACE	Chao1		
SRTS-1	6130	2722	7848	5455	7.2869	72.89
HN35-1	5500	2615	8863	5866	7.3031	69.20
SRTS-2	5596	2621	8510	5901	7.2914	69.94
HN35-2	5063	2233	7561	4840	7.1371	72.11
SRTS-3	5120	2284	7264	5074	7.1747	71.70
HN35-3	7418	3098	9602	6582	7.3845	74.31
SRTS-4	4518	2054	6376	4237	7.0772	71.51
HN35-4	5387	2624	9813	6324	7.3845	67.77
SRTS-5	6133	2768	8092	5880	7.3616	71.92
HN35-5	5254	2480	7742	5131	7.2727	70.14

^a^Number of sequences for sample.

^b^Calculated with DOTUR at the 3% distance level.

**Table 2:** Phylogenetic classification of genus for the sequences retrieved from rhizospheric soil samples of transgenic soybeans and nontransgenic soybeans during different growth stages (relative abundance > 0.18%).

Classification	Phylogenetic classification of clusters of sample (%)									
	SRTS-1	HN35-1	SRTS-2	HN35-2	SRTS-3	HN35-3	SRTS-4	HN35-4	SRTS-5	HN35-5
Gp6	12.48	8.89	9.72	4.50	2.99	15.37	2.08	11.10	11.19	5.06
*Sphingomonas*	9.14	7.35	7.90	3.40	2.54	8.29	3.32	7.43	6.13	5.10
Gp4	5.37	2.93	3.40	1.90	1.02	5.24	0.84	5.10	6.33	1.98
*Gemmatimonas*	2.14	2.31	2.11	1.66	1.80	2.32	2.26	2.26	3.49	2.36
Gp7	1.68	1.25	0.91	0.75	0.37	1.78	0.38	2.00	1.24	0.69
*Ohtaekwangia*	1.52	1.80	0.89	0.12	0.25	1.02	0.24	0.84	1.42	0.55
Gp16	1.24	1.04	1.18	0.73	0.53	1.46	0.62	1.21	0.73	0.74
*Kofleria*	1.00	0.69	0.61	0.47	0.45	0.92	0.62	0.84	0.75	0.67
*Azospirillum*	0.95	1.87	0.95	0.59	0.61	1.02	0.31	0.54	1.76	0.32
*Rubrobacter*	0.80	1.84	0.66	0.34	0.29	0.50	0.29	0.37	0.82	0.19
*Thermoleophilum*	0.70	0.45	0.36	0.41	0.53	0.31	0.27	0.33	0.31	0.38
*Phenylobacterium*	0.57	0.58	0.54	0.83	0.66	0.38	0.64	0.48	0.24	0.36
*Ramlibacter*	0.49	0.58	0.41	0.14	0.06	0.74	0.22	0.45	0.29	0.23
*Chitinophaga*	0.47	0.53	0.68	0.32	0.23	0.50	0.40	0.26	0.38	0.27
*Lysobacter*	0.47	0.45	0.55	0.22	0.16	0.36	0.11	0.37	0.62	1.50
*Flavitalea*	0.46	0.33	0.50	0.16	0.10	0.38	0.33	0.67	0.68	0.21
*Geminicoccus*	0.46	0.67	0.38	0.34	0.31	0.32	0.11	0.22	0.36	0.17
*Devosia*	0.44	0.71	0.52	0.71	0.78	0.26	1.37	0.52	0.29	0.80
*Microvirga*	0.38	1.25	0.45	0.14	0.25	0.36	0.13	0.37	0.55	0.29
*Adhaeribacter*	0.36	0.58	0.36	0.02	0.14	0.39	0.04	0.30	0.21	0.15
*Bauldia*	0.33	0.45	0.38	0.08	0.23	0.24	0.20	0.07	0.29	0.08
*Streptophyta*	0.29	0.15	0.21	0.61	0.98	0.05	0.53	0.17	0.13	0.36
*Dyella*	0.28	0.18	0.20	0.97	0.66	0.03	0.66	0.19	0.08	0.40
*Solirubrobacter*	0.28	0.22	0.27	0.34	0.53	0.11	0.77	0.28	0.08	0.40
*Roseomonas*	0.26	0.35	0.36	0.61	1.25	0.20	0.89	0.28	0.15	0.74
*Ralstonia*	0.24	0.58	0.36	1.17	0.96	0.13	0.55	0.17	0.08	0.95
*Dongia*	0.24	0.20	0.20	0.10	0.06	0.44	0.20	0.17	0.47	0.29
*Steroidobacter*	0.24	0.15	0.38	0.16	0.23	0.38	0.11	0.26	0.29	0.36
*Aquicella*	0.23	0.11	0.16	0.28	0.27	0.18	0.20	0.22	0.15	0.17
*Mesorhizobium*	0.21	0.18	0.16	0.91	0.61	0.16	0.55	0.32	0.08	0.65
*Nocardioides*	0.20	0.31	0.14	0.26	0.27	0.16	0.66	0.24	0.21	0.27
*Lactobacillus*	0.18	0.18	0.23	0.63	0.49	0.04	0.64	0.43	0.10	0.27
*Psychrobacter*	0.18	0.13	0.29	0.40	0.39	0.04	0.35	0.07	0.08	0.49
*Hyphomicrobium*	0.18	0.27	0.34	0.75	0.35	0.11	0.64	0.24	0.28	0.38
